# Utility of the Shortness of Breath in Daily Activities Questionnaire (SOBDA-Q) to Detect Sedentary Behavior in Patients with Chronic Obstructive Pulmonary Disease (COPD)

**DOI:** 10.3390/jcm12124105

**Published:** 2023-06-17

**Authors:** Yoshikazu Yamaji, Tsunahiko Hirano, Hiromasa Ogawa, Ayumi Fukatsu-Chikumoto, Kazuki Matsuda, Kazuki Hamada, Shuichiro Ohata, Ryo Suetake, Yoriyuki Murata, Keiji Oishi, Maki Asami-Noyama, Nobutaka Edakuni, Tomoyuki Kakugawa, Kazuto Matsunaga

**Affiliations:** 1 Department of Respiratory Medicine and Infectious Disease, Graduate School of Medicine, Yamaguchi University, Ube 755-8505, Japan; yyamaji@yamaguchi-u.ac.jp (Y.Y.); tsuna@yamaguchi-u.ac.jp (T.H.); chiku05@yamaguchi-u.ac.jp (A.F.-C.); k0m1a2t8s1u1d2a1@gmail.com (K.M.); khamada@yamaguchi-u.ac.jp (K.H.); j015ebponyou@gmail.com (S.O.); rsuetake@yamaguchi-u.ac.jp (R.S.); yomurata@yamaguchi-u.ac.jp (Y.M.); ohishk@yamaguchi-u.ac.jp (K.O.); noyamama@yamaguchi-u.ac.jp (M.A.-N.); edakuni@yamaguchi-u.ac.jp (N.E.); 2 Department of Occupational Health, Graduate School of Medicine, Tohoku University, Sendai 980-8575, Japan; ogawa-hiro@med.tohoku.ac.jp; 3 Department of Pulmonology and Gerontology, Graduate School of Medicine, Yamaguchi University, Ube 755-8505, Japan; tomoyukikakugawa@gmail.com

**Keywords:** sedentary behavior, physical activity, patient-reported outcome measures (PROMs), chronic obstructive pulmonary disease (COPD)

## Abstract

Sedentary behavior has been shown to be an independent predictor of mortality in patients with chronic obstructive pulmonary disease (COPD). However, physicians have difficulty ascertaining patients’ activity levels because they tend to avoid shortness of breath. The reformed shortness of breath (SOB) in the daily activities questionnaire (SOBDA-Q) specifies the degree of SOB by measuring low-intensity activity behavior in everyday living. Therefore, we aimed to explore the utility of the SOBDA-Q in detecting sedentary COPD. We compared the modified Medical Research Council dyspnea scale (mMRC), COPD assessment test (CAT), and SOBDA-Q with physical activity levels (PAL) in 17 healthy patients, 32 non-sedentary COPD patients (PAL ≥ 1.5 METs·h), and 15 sedentary COPD patients (PAL < 1.5 METs·h) in this cross-sectional study. CAT and all domains of the SOBDA-Q in all patients are significantly correlated with PAL, even after adjusting for age. The dietary domain has the highest specificity, and the outdoor activity domain has the highest sensitivity for detecting sedentary COPD. Combining these domains helped determine patients with sedentary COPD (AUC = 0.829, sensitivity = 1.00, specificity = 0.55). The SOBDA-Q is associated with PAL and could be a useful tool for determining patients with sedentary COPD. Moreover, eating and outing inactivity claims reflect sedentary behavior in patients with COPD.

## 1. Introduction

Shortness of breath (SOB) on exertion is one of the most important symptoms and is said to accompany exercise intolerance and reduce physical activity in patients with chronic obstructive pulmonary disease (COPD) [1,2]. Patients with symptomatic COPD spend less time walking and standing and, consequently, tend to be sedentary compared to healthy patients [3]. Recent studies show that sedentary behavior is an independent predictor of mortality in patients with COPD [4,5]. However, it is difficult for physicians to identify sedentary behavior in patients using a standard questionnaire because patients with a sedentary lifestyle are more likely to avoid the discomfort of feeling SOB on exertion and tend to underreport their symptoms. Therefore, in a clinical setting, we subjectively assessed sedentary behavior as physical activity (PA) using questionnaires and objectively assessed PA using pedometers and accelerometers [6]. Although conventional PA-related questionnaires are inexpensive and easy to apply to patients, they are reported to be less sensitive than accelerometers, especially when assessing low-intensity physical activity, including sedentary behavior [6]. This could arise from inaccurate perception, recall of information by the subject, questionnaire design, age, and the cognitive ability of the patient [6]. The University of California, San Diego Shortness of Breath questionnaire (UCSD SOBQ) [7] is a specific daily life activity questionnaire that uses the verbal rating scale. We devised the SOB in the daily activities questionnaire (SOBDA-Q), which can detect detailed situations in which patients with COPD experience SOB in their daily lives. Therefore, we hypothesized that the SOBDA-Q could be a useful tool for detecting sedentary behavior in patients with COPD.

## 2. Materials and Methods

### 2.1. Study Subjects

This is a cross-sectional study. We recruited 17 healthy patients and 47 patients with COPD who were treated at Yamaguchi University Hospital between January 2017 and November 2020. Healthy patients were defined as those with no respiratory, cardiovascular, or musculoskeletal diseases that interfered with their daily lives. COPD was diagnosed by a respiratory physician in accordance with the Global Initiative for Chronic Obstructive Lung Disease (GOLD) guidelines and treated according to the GOLD guidelines [1]. Patients with COPD were stable and had no exacerbations for at least three months before the study. Patients with other pulmonary diseases, such as interstitial lung disease and bronchiectasis, or those with restricted physical activity due to complications, such as cardiac or neuromuscular disease, were excluded. The study protocol was explained to healthy patients and COPD patients, and written informed consent was obtained from all patients. This study was approved by the local ethics committee of Yamaguchi Medical University (IRB number:H27-204).

### 2.2. Evaluation of Physical Activity

An Active Style Pro HJA-750C^®^ (Omron Healthcare Co., Ltd., Kyoto, Japan) with a triaxial acceleration sensor was attached to the waist, and physical activity was recorded continuously for 2 weeks. We measured the metabolic equivalent (METs) values and calculated the physical activity level (PAL) by multiplying the activity METs by the duration of the activity in hours (MET·h/day), as previously reported [8,9]. To obtain typical physical activity data, we collected values from weekdays without rain during the two weeks. The first and last days of recording were excluded because the data for one day were unavailable. Based on the 2011 Compendium of Physical Activity [10], we defined PAL < 1.5 METs as sedentary behavior [11], 1.6–2.9 METs as light-intensity activities [12], 3–5.9 METs as moderate-intensity activities, and ≥6 METs as vigorous-intensity activities. METs for various daily activities included approximately 1.3 METs for sitting and quietly watching TV, 2.0 METs for cooking and washing, 3.0 METs for walking the dog, 4.0 METs for climbing stairs, and 6.0 METs for bicycling. In addition, the American College of Sports Medicine and the American Heart Association recommend that older adults perform moderate-intensity (3 METs to 6 METs) aerobic exercise for at least 0.5 h five days a week [13]. Therefore, we considered patients with PAL < 1.5 METs·h as those with sedentary behavior [14]. In this study, we analyzed COPD groups with a PAL cutoff of <1.5 METs·h.

### 2.3. Assessment of Questionnaire Patient-Reported Outcome Measures (PROMs)

We used the modified Medical Research Council (mMRC) dyspnea scale, COPD assessment test (CAT), and SOBDA-Q as PROMs. The SOBDA-Q is a composite SOB questionnaire comprising 22 items organized into six domains of daily living (morning, dietary, indoor activity, outdoor activity, recreation, and night-time activity) (Table S1). The SOBDA-Q score was set using a scale with a minimum of one point (I do not do it because I feel short of breath) and a maximum of six points (I’m doing as usual without adjusting. I don‘t feel short of breath). Activity items that were not originally performed were defined as “no evaluation.” We calculated the average score for every domain except for the absence of evaluation. For example, if you scored 4 points for brushing teeth, 5 points for changing clothes, and 4 points for defecation/urination in the morning domain, the score for the morning domain would be calculated as (4 + 5 + 4)/3 items = 4.33 points (Table S2). This SOBDA-Q was devised and prepared by Dr. Hiromasa Ogawa, Tohoku University Hospital, with permission from the authors of the University of California, San Diego Shortness of Breath questionnaire (UCSD SOBQ) [7]. Trained research assistants administered the Japanese versions of the mMRC, CAT, and SOBDA-Q to participants.

### 2.4. Assessment of Pulmonary Function

Pulmonary function was assessed using a dry-rolling seal spirometer (CHESTAC-8800; Chest Co., Tokyo, Japan) according to the American Thoracic Society/European Respiratory Society recommendations [15].

### 2.5. Statistical Analysis

Data are shown as mean ± standard deviation. The chi-square test or Fisher’s exact test was used to compare the categorical data. Wilcoxon’s rank sum test was used to compare nonparametric data between each group. Spearman’s rank correlation and multiple linear regression analyses using the least-squares method were performed to analyze the correlation between PAL and PROMs. We analyzed sensitivity versus specificity using the area under the curve (AUC) and found a cutoff value for PROMs that could detect COPD patients with PAL of <1.5 METs·h. Statistical analyses were performed using JMP Pro^®^, version 15.0.0 (SAS Institute, Inc., Cary, NC, USA). Statistical significance was set at *p* < 0.05.

## 3. Results

### 3.1. Patient Characteristics

The baseline patient characteristics are shown in Table 1. This study included 17 healthy patients, 32 non-sedentary patients with COPD, and 15 sedentary patients with COPD. A total of 43 patients with COPD (91.5%) were classified as GOLD stages 1 and 2; 17 patients with COPD (36.2%) had a CAT score <10 points; and 35 patients with COPD (74.5%) had mMRC grades 0 and 1. Compared to healthy patients, patients with COPD were significantly more likely to be male, older, and to have a higher smoking status, CAT score, mMRC grade, and severe airflow limitation (*p* < 0.0001, *p* < 0.05, *p* < 0.0001, *p* < 0.0001, *p* < 0.05, and *p* < 0.05, respectively).

### 3.2. Comparison of the Physical Activity among Patients

A comparison of PAL, duration of physical activity, and total number of steps among the patients is shown in Table 2. PAL, duration of physical activity at all activity intensities, and the total number of steps are significantly lower in sedentary patients with COPD across all groups. In particular, the duration of ≥2 METs in sedentary patients with COPD is significantly reduced to about half of that in non-sedentary patients with COPD (≥2 METs: 208.5 min vs. 93.8 min, *p* < 0.0001), and the duration of ≥3 METs and ≥4 METs in sedentary patients with COPD are significantly reduced to about one-quarter of that in non-sedentary patients with COPD (≥3 METs: 55.2 min vs. 14.7 min, *p* < 0.0001; ≥4 METs: 12.2 min vs. 2.7 min, *p* < 0.0001, respectively).

### 3.3. Comparison of PROMs among Patients

Table 3 shows a comparison of the mean scores for each PROMs among the patients. Significantly higher mMRC and CAT scores and lower SOBDA-Q scores in all domains are observed in sedentary patients with COPD. In particular, the average scores in the morning, dietary, outdoor activity, recreation, and night-time activity domains are significantly lower in sedentary patients with COPD to non-sedentary patients with COPD.

### 3.4. Correlation between PROMs and Physical Activity

The correlations between PROMs and PAL are presented in Table 4. MMRC and CAT show a significant moderate negative correlation with PAL in univariate analysis. All SOBDA-Q domains show significant positive correlations with PAL in the univariate analysis. The linear regression model adjusted for age using the least-squares method shows that the CAT and all SOBDA-Q domains have significant independent associations with PAL.

### 3.5. The Relationship between Quartiles of PAL and PROMs

**Figure 1:**
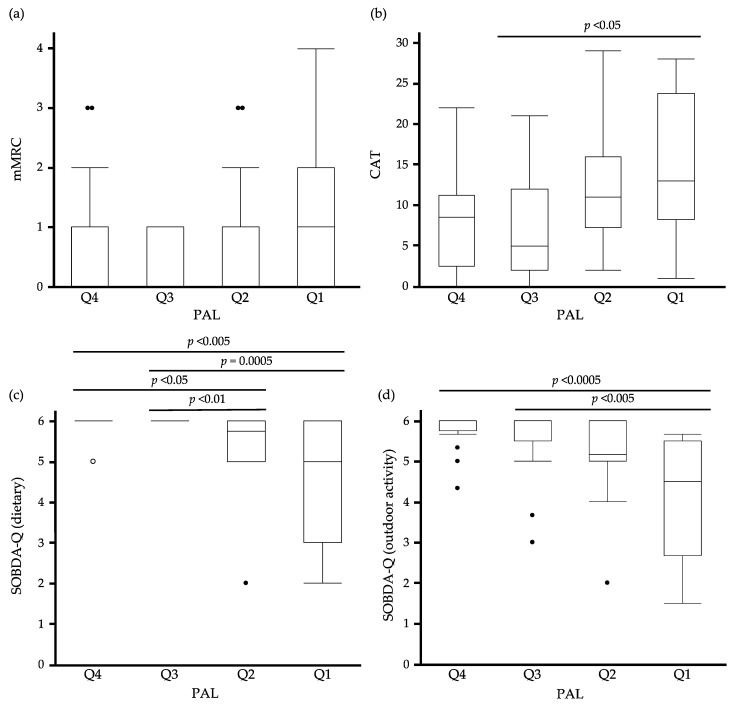
The relationship between quartiles of PAL and PROMs. (**a**) Box and whisker plots of PAL (quartiles) versus (**a**) mMRC, (**b**) CAT, (**c**) SOBDA-Q (dietary), and (**d**) SOBDA-Q (outdoor activity), where mMRC, CAT, and SOBDA-Q are represented by PAL (quartiles). Data are presented as the median and interquartile range (box), with minimum and maximum values (whiskers). Data beyond the end of the whiskers are called “outlying” points and are plotted individually. PROMs, patient-reported outcome measures; open circles, healthy patients; closed circles, COPD patients. Data were analyzed using the Wilcoxon test.

The relationship between quartiles of PAL (Q1: <1.6 METs·h, Q2: 1.6–3.0 METs·h, Q3: 3.0–4.5 METs·h, Q4: >4.5 METs·h) and mMRC, CAT, dietary, and outdoor activity domains of SOBDA-Q are shown in Figure 1. Significant differences are observed between the median values of Q1 and Q3 for CAT (13 vs. 5, *p* < 0.05). Similarly, the dietary domain shows significant differences between the median values of Q1, Q3, and Q4 (5 vs. 6, *p* = 0.0005, 5 vs. 6, *p* < 0.005, respectively) as well as between the median values of Q2 and Q3 or Q4 (5.75 vs. 6, *p* < 0.01, 5.75 vs. 6, *p* < 0.05, respectively). The outdoor activity domain shows significant differences between the median values of Q1, Q3, and Q4 (4.5 vs. 6, *p* < 0.005, 4.5 vs. 6, *p* < 0.0005, respectively).

### 3.6. The Diagnostic Ability of PROMs to Predict Sedentary Patients with COPD

**Figure 2:**
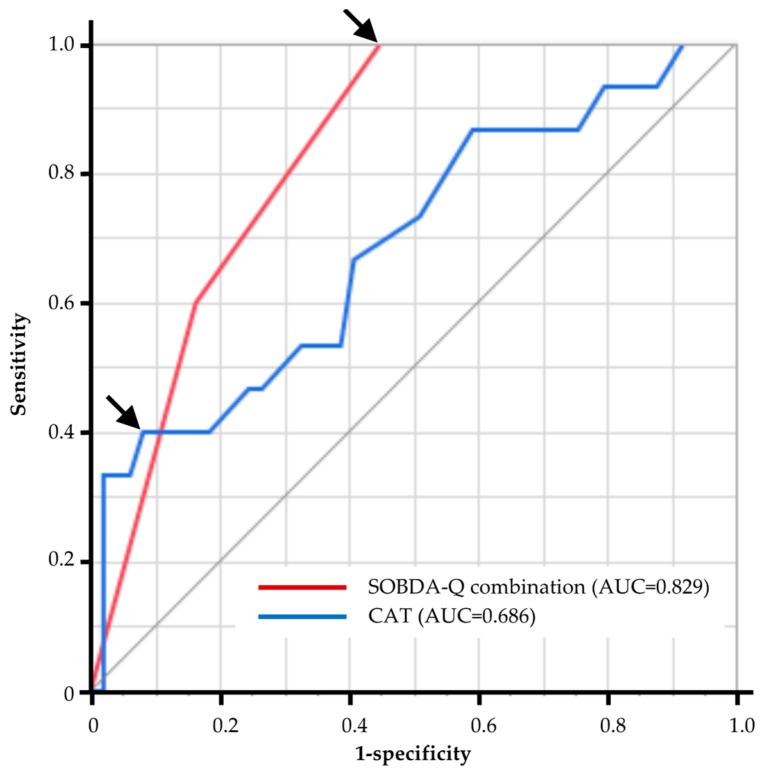
Diagnostic ability of CAT and SOBDA-Q combination to predict sedentary COPD. CAT, COPD assessment test; SOBDA-Q, Shortness of Breath in Daily Activities questionnaire; AUC, area under the curve. The arrow indicates the cut-off point.

We assessed the diagnostic ability of PROMs to predict sedentary patients with COPD using receiver operating characteristics (ROC) analysis (Table 5). The outdoor activity domain (cut off points ≤ 5.667) shows the highest area under the curve (AUC), and the dietary domain (cut off points ≤ 5.5) shows the second highest. Figure 2 shows the ROC curves for CAT (cut off points ≥ 21) and the SOBDA-Q combination for the two domains (outdoor activity and dietary). The ROC analysis of the SOBDA-Q combination for predicting COPD in sedentary patients has an AUC of 0.829, a sensitivity of 1.00, and a specificity of 0.55.

### 3.7. The Relationship between Stratified CAT, SOBDA-Q Combination, and PAL

**Figure 3:**
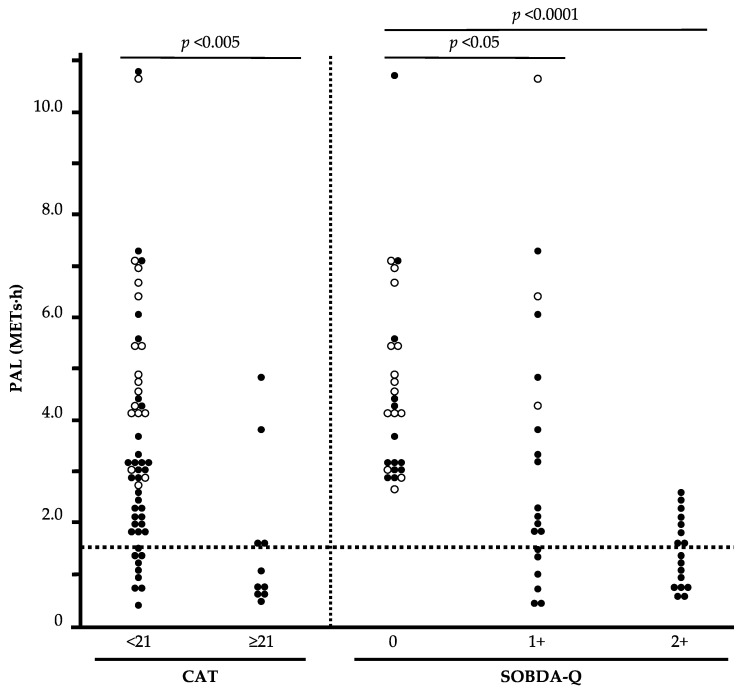
The relationship between stratified CAT, SOBDA-Q combination, and physical activity. CAT, COPD assessment test; SOBDA-Q, Shortness of Breath in Daily Activities questionnaire; open circles: healthy patients; closed circles: COPD patients. The dotted line represents 1.5 METs·h. Data were analyzed with the Wilcoxon test.

Using the cutoff values obtained from ROC analysis, a scatter plot of the relationship between CAT, SOBDA-Q combination, and PAL is shown in Figure 3. Compared to the CAT score <21 group, the CAT score ≥21 group shows significantly less physical activity (*p* < 0.005). Compared with both negative groups of the SOBDA-Q combination (two domains negative), at least one domain-positive group (one domain positive or two domains positive) shows significantly less physical activity (*p* < 0.05, *p* < 0.0001, respectively). In particular, there are no sedentary patients with COPD who test negative in the two domains of the SOBDA-Q combination.

## 4. Discussion

We demonstrate that the newly devised SOBDA-Q significantly correlates with physical activity levels. Moreover, the combination of dietary- and outdoor-activity-related SOB can accurately identify patients with sedentary COPD.

The International Physical Activity questionnaire (IPAQ) [16] and its modified version, the Global Physical Activity questionnaire (GPAQ) [17], are representative physical activity questionnaires. They are not applied to patients with COPD but to the general adult population. They do not have sufficient sensitivity to detect low-intensity physical activity reduction [18], which is a feature of patients with COPD [3]. Meanwhile, the Minnesota Leisure Time Physical Activity questionnaire (Minnesota LTPA Questionnaire), the Baecke Questionnaire of Habitual Physical Activity, and the Physical Activity Scale in the Elderly (PASE) questionnaire are more valid in the older and other populations [6,19,20,21]. However, since many patients with COPD limit their daily activities to avoid experiencing dyspnea and underestimate the feeling of dyspnea by patients themselves [22], it is difficult for physicians to accurately analogize physical activity, especially daily physical activity, using the above self-reported questionnaires. We show that in the conventional subjective SOB evaluation questionnaire, the mMRC is poorly correlated with the objectively measured PAL and that the mMRC does not increase in a PAL-dependent manner (Table 4 and Figure 1). In short, the simple SOB questionnaire has limitations in assessing the physical activity of patients with COPD who tend to avoid daily activities [23,24,25].

CAT also did not have sufficient power to capture physical activity as sensitively as SOBDA-Q in this study. CAT includes major symptoms in patients with COPD [26]; however, some questions, such as cough, sputum, and sleep status, are not associated with physical activity. Furthermore, CAT is formatted as a semantic differential six-point scale [26], and it may require higher cognitive demands from participants owing to the abstract level of interpretation between the end-label representations [27]. In other words, the interpretation of each person’s end-label scores differs depending on how each person sets the maximum end-label value. On the other hand, SOBDA-Q can reflect wide-intensity activities in various life situations and specifically determine the latent behavior that saves daily activities due to SOB. Therefore, as shown in Table 3, SOBDA-Q shows significant differences in mean scores not only between HP and sedentary patients with COPD, but also between non-sedentary patients and sedentary patients with COPD. We consider this detection ability to be a strength of SOBDA-Q. Additionally, SOBDA-Q indicates the degree of breathlessness in specific terms, making it easier for participants to choose their options without hesitation. Hence, as shown in Table 4 and Figure 1, SOBDA-Q may have resulted in more PAL-dependent changes than CAT.

Furthermore, within each SOBDA-Q domain, the dietary and outdoor activity domains are more important for detecting sedentary COPD (Table 4 and Table 5). Meal-induced dyspnea in patients with COPD is thought to be related to irregular breathing caused due to chewing and swallowing [28,29], abdominal distension after eating [30], and upper extremity exertion during eating [31,32]. In addition, the causes of an outdoor-activity-related SOB in patients with COPD are thought to be related to increased respiratory rate and inspiratory time/total time ratio (Tin/Ttot) and end-expiratory load during a conversation [33,34,35]. However, the causal relationship is unclear, and further analysis is required to understand this mechanism.

The specific SOBDA-Q combination had high sensitivity (1.00) for detecting sedentary COPD (Figure 2). This means that asking patients with COPD questions related to dietary and outdoor activities would be useful as a screening tool for sedentary behavior. Although approximately 90% of the patients with COPD in this study had GOLD stages 1 and 2, approximately 30% had already been sedentary. This is consistent with previous studies in which COPD patients with GOLD stages 1 or 2 had reduced physical activity [3]. These results suggest that our SOBDA-Q is useful for detecting sedentary behavior in early-stage COPD and for initiating early intervention for COPD.

Regarding the clinical application of SOBDA-Q, Hirano et al. demonstrated that using SOBDA-Q as a guide for the assistive use of short-acting β2 agonists (SABA) can increase PAL in patients with COPD [36]. Using the SOBDA-Q as a communication tool in daily medical practice, medical staff (doctors, nurses, etc.) can recognize and understand patient-specific breathlessness situations and circumstances. Medical staff will be able to instruct patients with COPD in situations where they need assistive use of SABA. Patients with COPD can also access this tool to understand when and how to use assistive use of SABA. In particular, the SOBDA-Q can identify unrecognized inactivity, which has been restricted owing to potential SOB in daily life. This concept coincides with previous studies showing that behavioral change counseling is needed to improve physical inactivity [37]. We believe that the SOBDA-Q can be an interactive communication tool between medical staff and patients with COPD that promotes behavioral change.

However, there are several limitations in generalizing the results of this study. First, the sample size was small, and there was a difference in the ratio of male to female participants between healthy patients and patients with COPD. Some studies have reported sex differences in breathlessness and other symptoms in patients with COPD [38,39], which may have affected the results of this study. In addition, multivariate analysis including sex, smoking status, and pulmonary function as confounding factors could not be performed in this study because of lack of statistical power due to the small sample size. In the future, we will examine this issue with a large sample size. Second, many patients had early GOLD stage. As there may be differences in symptoms and physical activity according to the severity of airflow obstruction [40], further studies are needed to verify this by recruiting more patients with more severe stages. Third, the results were evaluated using the average score for each SOBDA-Q domain. Using the average score, we were able to compare the SOB in each daily lifestyle; however, some patients did not perform all daily physical activities in the domains. Fourth, we did not compare SOBDA-Q to questionnaires other than the mMRC or CAT. Therefore, we cannot mention any differences from other questionnaires. We will consider further studies that incorporate other questionnaires in the future. Fifth, we did not perform a combination analysis of the SOBDA-Q as a questionnaire for identifying sedentary patients with COPD. Therefore, the present study did not examine the effect of SOBDA-Q in combination with CAT or mMRC. Further studies will be conducted to increase the sample size and examine the usefulness of combining the SOBDA-Q with other questionnaires.

## 5. Conclusions

The newly developed SOBDA-Q correlates significantly with objectively measured physical activity and has the potential to accurately identify patients with sedentary COPD.

## Figures and Tables

**Table 1:** Patient characteristics.

	HP (N = 17)	Non-Sedentary COPD (N = 32)	Sedentary COPD (N = 15)	*p* Value
Number, n (M/F)	4/13	32/0	14/1	<0.0001
Age (years)	61.1 ± 10.6	70.0 ± 8.0	70.9 ± 10.2	<0.05
BMI (kg/m^2^)	21.8 ± 3.5	22.6 ± 2.8	24.9 ± 4.1	n.s.
Smoking status (non/ex/cu)	15/2/0	0/24/8	0/9/6	<0.0001
Smoking history (pack years)	3.6 ± 11.0	45.2 ± 26.7	51.8 ± 22.0	<0.0001
GOLD (1/2/3/4)	-	16/13/3/0	5/9/1/0	-
CAT (<10/≥10)	17/0	12/20	5/10	<0.0001
mMRC(0/1/2/3/4)	13/4/0/0/0	16/9/2/5/0	5/5/4/0/1	<0.05
FEV_1_	2.38 ± 0.42	2.21 ± 0.56	1.90 ± 0.51	<0.05
%FEV_1_	106.8 ± 12.6	77.7 ± 18.7	70.4 ± 16.0	<0.0001

Data are shown as means ± standard deviation. Abbreviations: HP, healthy patients; COPD, chronic obstructive pulmonary disease; BMI, body mass index; non, non-smoker; ex, ex-smoker; cu, current smoker; GOLD, Global Initiative for Chronic Obstructive Lung Disease; CAT, COPD assessment test; mMRC, modified Medical Research Council; FEV_1_, forced expiratory volume in one second. *p*-value compared across all groups with the chi-square test or Fisher’s exact test or the Wilcoxon test.

**Table 2:** Comparison of the physical activity among patients.

	HP (N = 17)	Non-Sedentary COPD (N = 32)	Sedentary COPD (N = 15)	*p* Value
PAL (METs·h)	5.18 ± 1.95 ^a^	3.50 ± 1.99 ^a^	0.87 ± 0.33	<0.0001
≥1METs (min)	776.1 ± 151.4 ^b^	609.4 ± 180.9 ^c^	495.5 ± 222.3	<0.0005
≥2METs (min)	259.8 ± 79.8 ^a^	208.5 ± 87.2 ^a^	93.8 ± 50.8	<0.0001
≥3METs (min)	83.9 ± 33.4 ^a^	55.2 ± 31.3 ^a^	14.7 ± 5.6	<0.0001
≥4METs (min)	20.2 ± 10.9 ^a^	12.2 ± 14.6 ^a^	2.7 ± 2.0	<0.0001
Total number of steps	6315.5 ± 2284.8 ^a^	5224.7 ± 2860.0 ^a^	1714.6 ± 1755.8	<0.0001

Data are shown as means ± standard deviation. Abbreviations: HP, healthy patients; COPD, chronic obstructive pulmonary disease; PAL, physical activity level; METs: metabolic equivalents; *p*-value compared across all groups with Wilcoxon test. ^a^ *p* < 0.001, ^b^ *p* < 0.01, ^c^ *p* < 0.05, in reference to sedentary COPD.

**Table 3:** Comparison of PROMs among patients.

	HP (N = 17)	Non-Sedentary COPD (N = 32)	Sedentary COPD (N = 15)	*p* Value
mMRC	0.2 ± 0.4 ^b^	0.9 ± 1.1	1.1 ± 1.1	<0.05
CAT	4.1 ± 3.2 ^a^	11.7 ± 6.7	15.4 ± 9.1	<0.0001
SOBDA-Q				
Morning	5.9 ± 0.2 ^a^	5.5 ± 0.7 ^c^	4.4 ± 1.5	<0.001
Dietary	5.9 ± 0.2 ^a^	5.6 ± 0.8 ^b^	4.3 ± 1.6	<0.0005
Indoor activity	5.9 ± 0.3 ^b^	5.2 ± 1.0	4.3 ± 1.8	<0.05
Outdoor activity	6.0 ± 0.1 ^a^	5.1 ± 1.1 ^c^	3.8 ± 1.7	<0.0001
Recreation	5.6 ± 0.4 ^a^	4.8 ± 1.1 ^c^	3.7 ± 1.7	<0.0005
Night-time activity	5.9 ± 0.3 ^a^	5.4 ± 0.9 ^c^	4.3 ± 1.5	<0.001

Data are shown as means ± standard deviation. Abbreviations: HP, healthy patients; COPD, chronic obstructive pulmonary disease; mMRC, modified Medical Research Council; CAT, COPD assessment test; SOBDA-Q, shortness of breath in daily activities questionnaire. *p*-value compared across all groups with Wilcoxon test. ^a^ *p* < 0.001, ^b^ *p* < 0.01, ^c^ *p* < 0.05, in reference to sedentary COPD.

**Table 4:** Correlation between PROMs and physical activity in all patients.

Variables	Univariate Analysis		Multivariate Analysis	
	Correlation Coefficient (ρ)	*p* Value	Correlation Coefficient (F)	*p* Value
mMRC	−0.27	<0.05	0.25	n.s.
CAT	−0.42	<0.001	4.72	<0.05
SOBDA-Q				
Morning	0.48	<0.0001	5.46	<0.05
Dietary	0.6	<0.0001	5.5	<0.05
Indoor activity	0.44	<0.001	5.33	<0.05
Outdoor activity	0.62	<0.0001	6.28	<0.05
Recreation	0.44	<0.0005	4.23	<0.05
Night-time activity	0.44	<0.0005	5.04	<0.05

Univariate analysis was performed by Spearman’s rank correlation coefficient. For multivariate analysis, multiple linear regression analysis using the least-squares method were performed to adjust correlation of univariate analysis by age. Abbreviations: n.s., not statistically significant.

**Table 5:** Diagnostic ability in PROMs to predict sedentary patients with COPD.

Variables	AUC	Cut Off	Sensitivity	Specificity
mMRC	0.641	1	0.67	0.59
CAT	0.686	21	0.4	0.92
SOBDA-Q				
Morning	0.733	5	0.67	0.76
Dietary	0.759	5.5	0.67	0.8
Indoor activity	0.679	5	0.54	0.76
Outdoor activity	0.817	5.667	1	0.58
Recreation	0.755	4.667	0.67	0.76
Night-time activity	0.756	5.667	0.73	0.73

Abbreviations: mMRC, modified Medical Research Council; CAT, COPD assessment test; SOBDA, shortness of breath in daily activities questionnaire; AUC, area under the curve.

## Data Availability

The data analyzed during the current study are included in this article. Additional data are available from the corresponding author upon request.

## Supplementary Materials

The following supporting information can be downloaded at: https://www.mdpi.com/article/10.3390/jcm12124105/s1, Table S1: The Shortness of Breath in the Daily Activities questionnaire (SOBDA-Q); Table S2: The SOBDA-Q scoring method (example).
