# Supplementary material for: Utility of the Shortness of Breath in Daily Activities Questionnaire (SOBDA-Q) to Detect Sedentary Behavior in Patients with Chronic Obstructive Pulmonary Disease (COPD)
Source: J Clin Med. 2023 Jun 17;12(12):4105. doi: 10.3390/jcm12124105 (PMC10299225; doi:10.3390/jcm12124105)
Supplement: Supplementary file 1 [file jcm-12-04105-s001.zip › jcm-2401258-supplementary.pdf]

Supplementary Materials:

Table S1. The Shortness of Breath in the Daily Activities Questionnaire (SOBDA-Q).

		I do not do it because I feel short of breath.	I am doing moderately (slowly, rest)		I'm doing as usual without adjusting.			I don't do it originally, so I don't know if I feel short of breath.
			But I feel short of breath.	I don't feel short of breath because I adjust.	But sometimes I feel short of breath and need to rest.	I will not feel short of breath unless I overdo it.	I don't feel short of breath.	
**score**		**1**	**2**	**3**	**4**	**5**	**6**	**No evaluation**
**morning**	Brushing teeth, washing face, shaving and hairdressing							
	changing clothes							
	Defecation/urination							
**dietary**	Eating							
	Meal preparation, cooking and cleanup							
**indoor activity**	Hanging the laundry /taking the laundry							
	Tidying up room / Cleaning up room							

	Lifting luggage							
**outdoor activity**	Talking while standing							
	Gardening (weeding, watering)							
	Washing the car							
**recreation**	Going out on foot (walking, shopping)							
	Going out by bicycle (cycling, shopping))							
	Walking on level ground at my own pace							
	Walking on level ground with people of the same age							
	Taking care of animals							
	Playing with children and grandchildren							
	Going up the slope							
	Going up the stairs							
**nighttime activity**	Taking a shower or bath							
	Washing your hair and body							
	Making beds							

Table S2. The SOBDA-Q scoring method (example).

		I do not do it because I feel short of breath.	I am doing moderately (slowly, rest)		I'm doing as usual without adjusting.			I don't do it originally, so I don't know if I feel short of breath.
			But I feel short of breath.	I don't feel short of breath because I adjust.	But sometimes I feel short of breath and need to rest.	I will not feel short of breath unless I overdo it.	I don't feel short of breath.	
**score**		**1**	**2**	**3**	**4**	**5**	**6**	**No evaluation**
**morning**	Brushing teeth, washing face, shaving and hairdressing				○			
	changing clothes					○		
	Defecation/urination				○			
